# Prognostic Value of Excision Repair Cross-Complementing mRNA Expression in Gastric Cancer

**DOI:** 10.1155/2018/6204684

**Published:** 2018-10-17

**Authors:** Shan-Shan Luo, Xi-Wen Liao, Xiao-Dong Zhu

**Affiliations:** ^1^Department of Gastrointestinal Surgery, Affiliated Tumor Hospital of Guangxi Medical University, Nanning 530021, Guangxi Zhuang Autonomous Region, China; ^2^Department of Hepatobiliary Surgery, The First Affiliated Hospital of Guangxi Medical University, Nanning 530021, Guangxi Zhuang Autonomous Region, China; ^3^Department of Radiation Oncology, Affiliated Tumor Hospital of Guangxi Medical University, Cancer Institute of Guangxi Zhuang Autonomous Region, Nanning 530021, Guangxi Zhuang Autonomous Region, China

## Abstract

Except for excision repair cross-complementing 1 (ERCC1), mRNA expression of the remaining ERCC genes has not been investigated in the prognosis of gastric cancer (GC). The present study aimed to explore the mRNA expression and prognostic values of each member of the ERCC family in GC patients by using the Kaplan–Meier (KM) plotter tool. The details of each ERCC family member were entered into a database and GC patients were separated into high and low expression to draw survival plots using the KM plotter. In the present study, we observed that high expression of ERCC1 mRNA was significantly associated with longer overall survival (OS) for all GC patients (hazard ratio [HR]=0.77, 95% confidence intervals [CI]=0.63–0.95, P=0.016) compared with low expression. High expression of ERCC4 and ERCC6 mRNA indicated a worse OS for all GC patients (HR=1.28, 95% CI=1.02–1.6, P=0.035 and HR=1.25, 95% CI=1.02–1.54, P=0.029, respectively) and especially for patients with intestinal-type GC (HR=1.87, 95% CI=1.26–2.79, P=0.0018 and HR=1.62, 95% CI=1.04–2.54, P=0.033, respectively). High ERCC8 mRNA expression indicated a worse OS for all GC patients (HR=1.34, 95% CI=1.02–1.76, P=0.034) and especially for patients with diffuse-type GC (HR=2.25, 95% CI=1.36–3.75, P=0.0013). In conclusion, our findings indicate that ERCC4, ERCC6, and ERCC8 may be potential biomarkers for GC prognosis and may serve as potential therapeutic targets for GC. However, these findings still need further verification.

## 1. Introduction

Gastric cancer (GC) is the fifth most common malignancy and results in the third leading cause of cancer-associated mortality universally. Overall, 951,600 new cases of GC and 723,100 deaths occurred in 2012 with the highest rates of incidence in Eastern Asia, Central and Eastern Europe, and South America [1]. Surgical treatment of GC has greatly improved in the last few decades and has become increasingly effective, but the age-standardized 5-year relative survival was only 27.4% in China in 2010 and 29% in the USA in 2009 [2, 3]. Moreover, because of tumor heterogeneity, the same TNM stage of GC can have a different therapeutic response and prognosis. Thus, an exploration of the molecular mechanisms of tumorigenesis, progression, therapeutic response, and prognosis, as well as the development of prognostic markers and targeted medicine, is urgently required.

The excision repair cross-complementing (ERCC) family includes ERCC1, ERCC2 (also known as XPD), ERCC3 (also known as XPB), ERCC4 (also known as XPF), ERCC5 (also known as XPG), ERCC6 (also known as CSB), and ERCC8 (also known as CSA). A previous study by Yao and his coworkers has found that the expression of ERCC1 was associated with survival time and chemotherapy regimens in GC [4]. Nevertheless, the results of these studies were inconsistent [5, 6]. It was reported that a polymorphism of ERCC2 was associated with a reduced response to chemotherapy and overall survival (OS) in GC patients receiving oxaliplatin treatment [7]. In colorectal cancer, ERCC2 overexpression failed to predict the survival time of patients receiving adjuvant chemotherapy [8], but the expression and prognosis ability of ERCC2 mRNA in GC are unreported. The mRNA expression of ERCC3 and its prognosis ability in GC is also unreported. Abolfazl et al. reviewed 33 case-control studies and found that polymorphisms of the ERCC5 gene are associated with susceptibility to GC [9]. The mRNA expression of the remaining ERCC genes has not been investigated in the prognosis of GC in previous studies.

The “Kaplan–Meier plotter” (KM plotter) database was generated using gene expression data and survival information downloaded from GEO (http://www.ncbi.nlm.nih.gov/geo/). Currently, the KM plotter is able to evaluate the effect of 54,675 genes on survival using 10,461 cancer samples. It includes 5,143 breast cancer, 1,816 ovarian cancer, 2,437 lung cancer, and 1,065 GC patients with a mean follow-up of 69 months. Therefore, the KM plotter is broadly used for the analysis of the clinical impact of individual genes on survival time in cancer patients, including GC. In the present study, we explored the mRNA expression and prognostic values of each member of the ERCC family in human GC patients via the KM plotter database.

## 2. Material and Methods

We used the Database for Annotation, Visualization and Integrated Discovery (DAVID, https://david.ncifcrf.gov/home.jsp; accessed March 1, 2018) v.6.8 to explore the Gene Ontology (GO) and Kyoto Encyclopedia of Genes and Genomes (KEGG) enrichment of ERCC genes [10]. We used the gene multiple association network integration algorithm (Gene MANIA; http://www.genemania.org/; accessed March 1, 2018) to structure gene–gene networks [11, 12] and used the Search Tool for the Retrieval of Interacting Genes/Proteins (STRING v.10.0; https://string-db.org/; accessed March 1, 2018) to structure protein–protein interaction (PPI) networks [13–15]. We constructed a coexpression heat map of ERCC mRNA by using the mRNA expression of GC tumor tissues from the GSE14210, GSE15459, and GSE51105 datasets. Microarray data were normalized according to manufacturer's instructions (GSE14210 [https://www.ncbi.nlm.nih.gov/geo/query/acc.cgi?acc=GSM355175], GSE15459 [https://www.ncbi.nlm.nih.gov/geo/query/acc.cgi?acc=GSM387816], and GSE51105 [https://www.ncbi.nlm.nih.gov/geo/query/acc.cgi?acc=GSM1238811]). We used Pearson's correlation coefficient to evaluate the coexpression correlation at the mRNA expression level. The coexpression heat map was structured using the* corrplot* package in the R 3.4.4 platform. We used the online database KM plotter via the website (http://kmplot.com/analysis/index.php?p=service&cancer=gastric) and the ERCC family details were submitted to the database. The GC patients were separated into high and low expression by the median, clinical parameters, including stage, stage TNM, Lauren classification, differentiation, treatment, and HER2 status, which were all used to draw the KM survival plots. Statistical analyses were performed by SPSS v.22.0 software (IBM Corp., Armonk, NY, USA). The results contain survival plot, hazard ratio (HR), 95% confidence intervals (CI), and log-rank P. A P value of <0.05 was considered to be statistically significant.

## 3. Results

**Figure 1:**
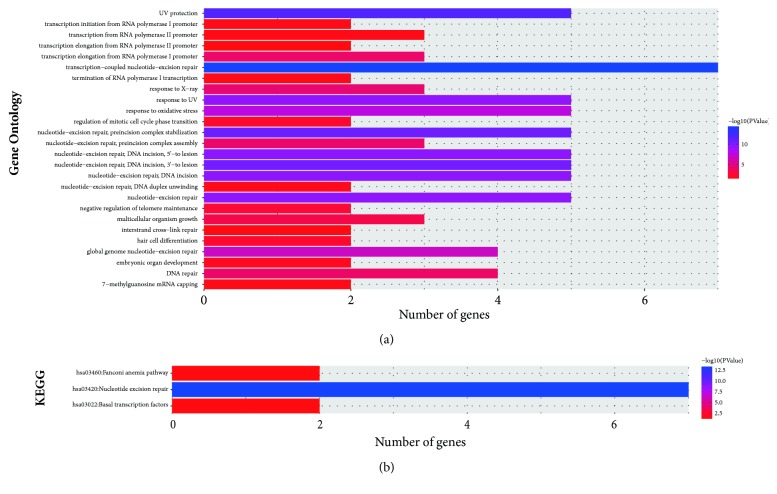
GO and KEGG analysis of ERCC genes. (a) GO enrichment analysis of ERCC genes. (b) KEGG enrichment analysis of ERCC genes.

The results of GO analysis are shown in Figure 1(a). The functions of the ERCC gene family include transcription elongation/initiation from RNA polymerase I/II promoter, nucleotide excision repair (NER), termination of RNA polymerase I transcription, DNA duplex unwinding, interstrand cross-link repair, and DNA repair. Analysis of KEGG suggested that the ERCC gene family's function was involved in hsa03420: NER and sa03022: basal transcription factors (Figure 1(b)).

**Figure 2:**
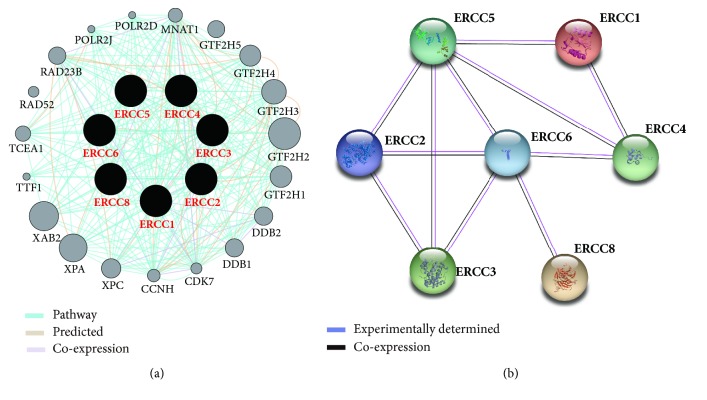
Gene and protein interaction networks of ERCC genes. (a) Gene multiple association network integration algorithm. (b) Protein–protein interaction networks.

**Figure 3:**
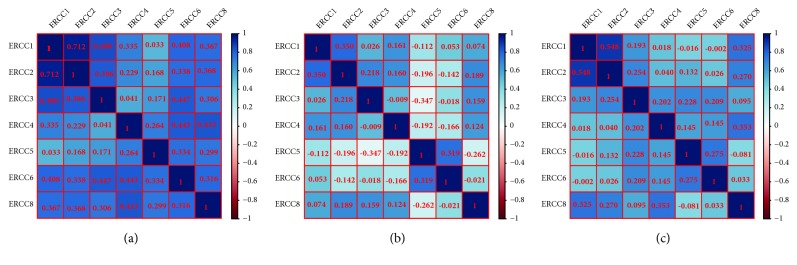
Coexpression heat map of ERCC genes with each other in the GSE14210cor dataset (a), GSE15459cor dataset (b), and GSE51105cor dataset (c). The numbers shown in red are the r-values of the Pearson correlation coefficient.

As shown in Figure 2, the analysis of gene and protein interaction networks revealed that the ERCC gene family and other relevant genes constructed a complex network with each other. Gene–gene interaction networks suggest that the ERCC gene family are coexpressed with each other (Figure 2(a)), and the PPI network data showed that ERCC directly or indirectly contacted each other (Figure 2(b)). In addition, there was coexpression of the ERCC genes in GC tumor tissues (Figures 3(a)–3(c)).

**Figure 4:**
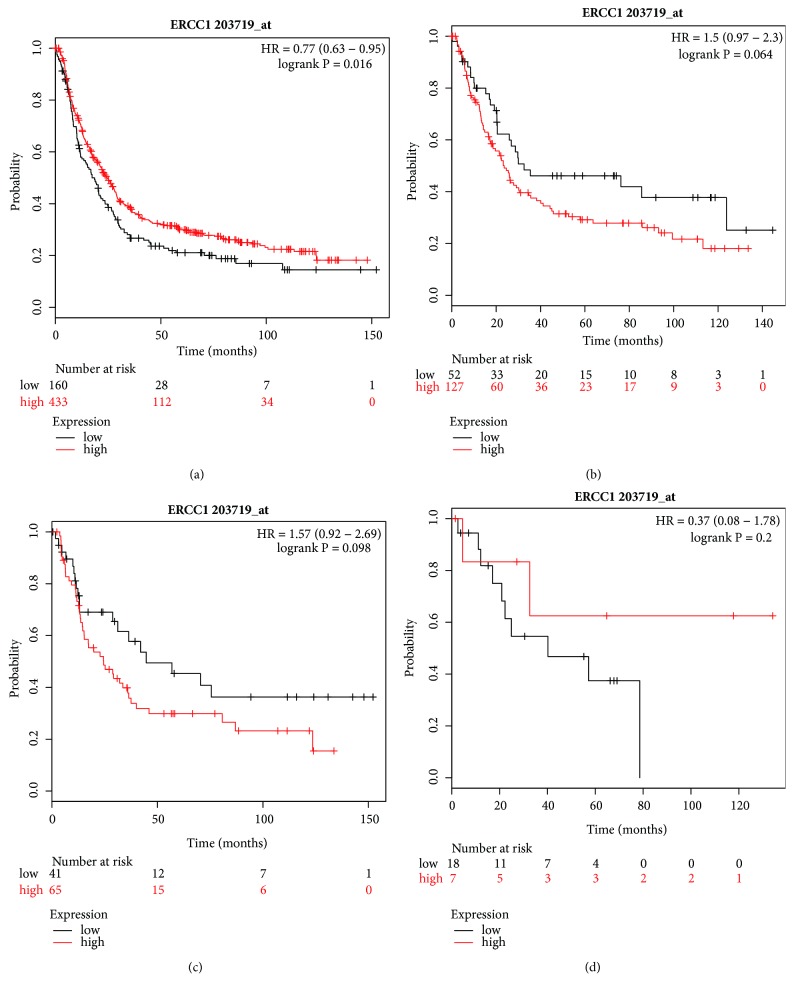
Prognostic value of* ERCC1* (203719_at) expression in Kaplan–Meier plotter tool. Overall survival curves are plotted for (a) all patients (n=593) and patients with (b) intestinal-type (n=179), (c) diffuse-type (n = 106), and (d) mixed-type (n = 25) gastric cancer.

Firstly, we analyzed the prognostic role of ERCC1 in mRNA expression. The Affymetrix ID of ERCC1 in the KM plotter was 203719_at and Figure 4 reveals the prognostic value of mRNA expression of ERCC1. The high expression of ERCC1 mRNA was significantly associated with longer OS for all GC patients (HR=0.77, 95% CI=0.63–0.95, P=0.016, Figure 4(a)), but for different Lauren classifications in GC patients, there was no impact on OS (Figures 4(b)–4(d)) compared with low expression.

**Figure 5:**
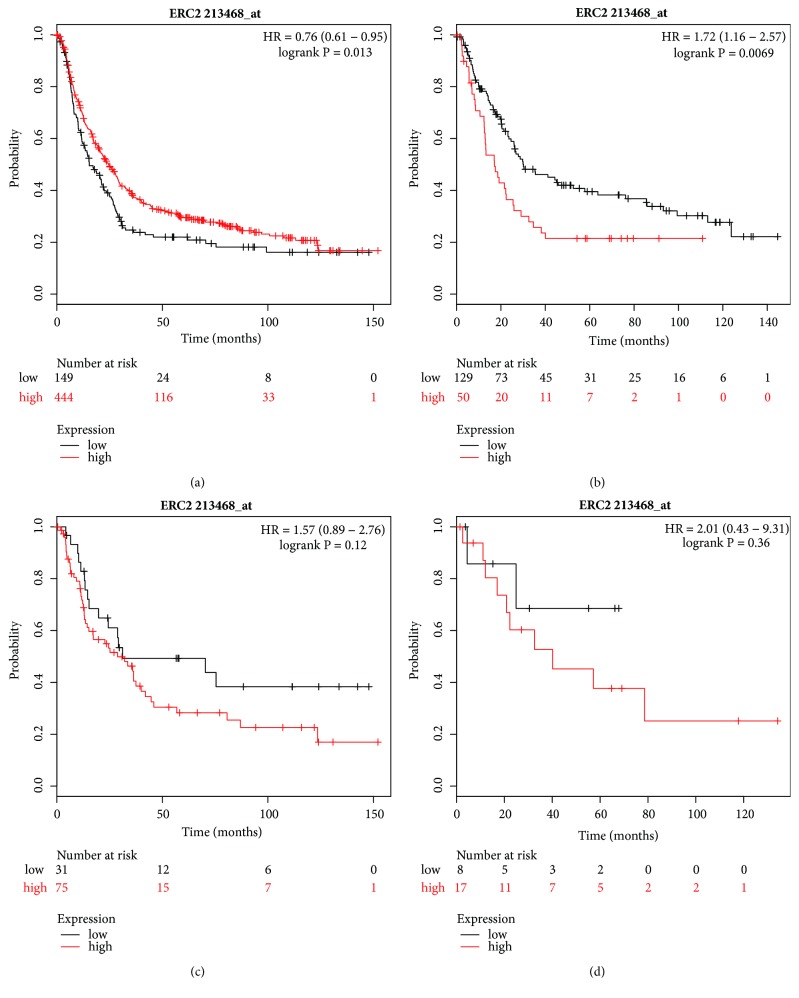
Prognostic value of* ERCC2* (213468_at) expression in Kaplan–Meier plotter tool. Overall survival curves are plotted for (a) all patients (n=593) and patients with (b) intestinal-type (n=179), (c) diffuse-type (n = 106), and (d) mixed-type (n = 25) gastric cancer.

The Affymetrix ID of ERCC2 was 213468_at. The high expression of ERCC2 mRNA was significantly associated with favorable OS for all GC patients (HR=0.76, 95% CI=0.61–0.95, P=0.013, Figure 5(a)). In contrast, it was markedly associated with poor OS for intestinal-type GC (HR=1.72, 95% CI=1.16–2.57, P=0.0069, Figure 5(b)) and there was no impact on OS for diffuse- and mixed-type GC patients (Figures 5(c) and 5(d)).

**Figure 6:**
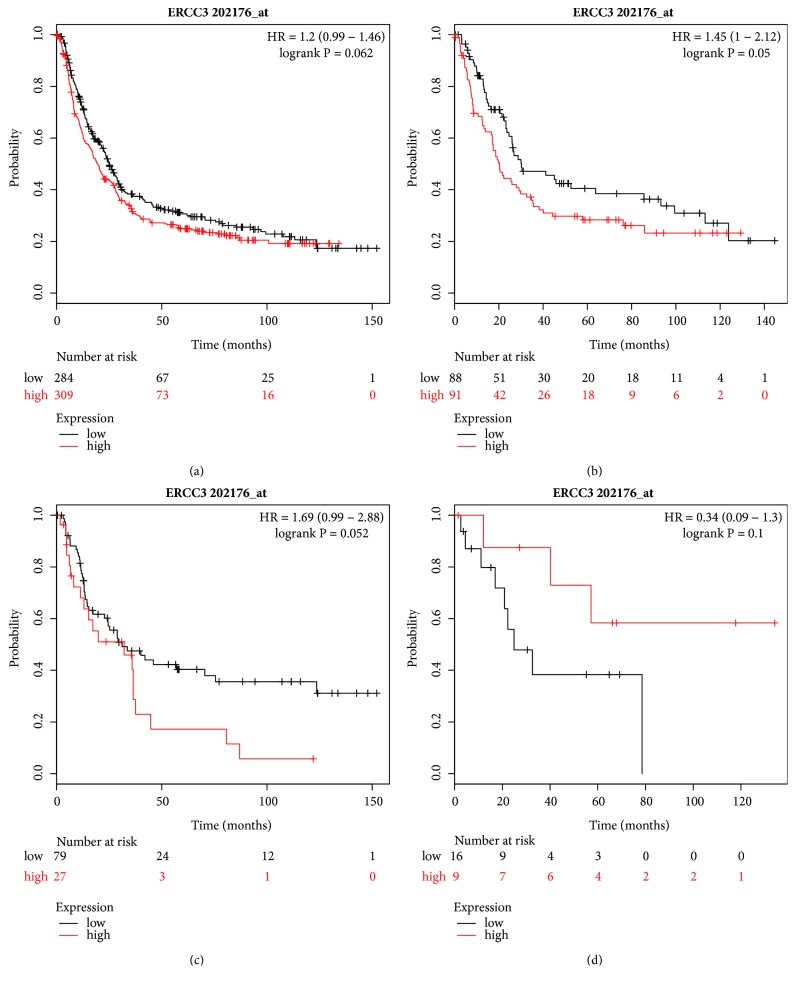
Prognostic value of* ERCC3* (202176_at) expression in the Kaplan–Meier plotter tool. Overall survival curves are plotted for (a) all patients (n=593) and patients with (b) intestinal-type (n=179), (c) diffuse-type (n = 106), and (d) mixed-type (n = 25) gastric cancer.

Figure 6 shows the prognosis of the mRNA expression of ERCC3 (Affymetrix ID: 202176_at). No relationship was revealed between ERCC3 mRNA expression and OS for all GC patients and those with different Lauren classification (Figures 6(a)–6(d)).

**Figure 7:**
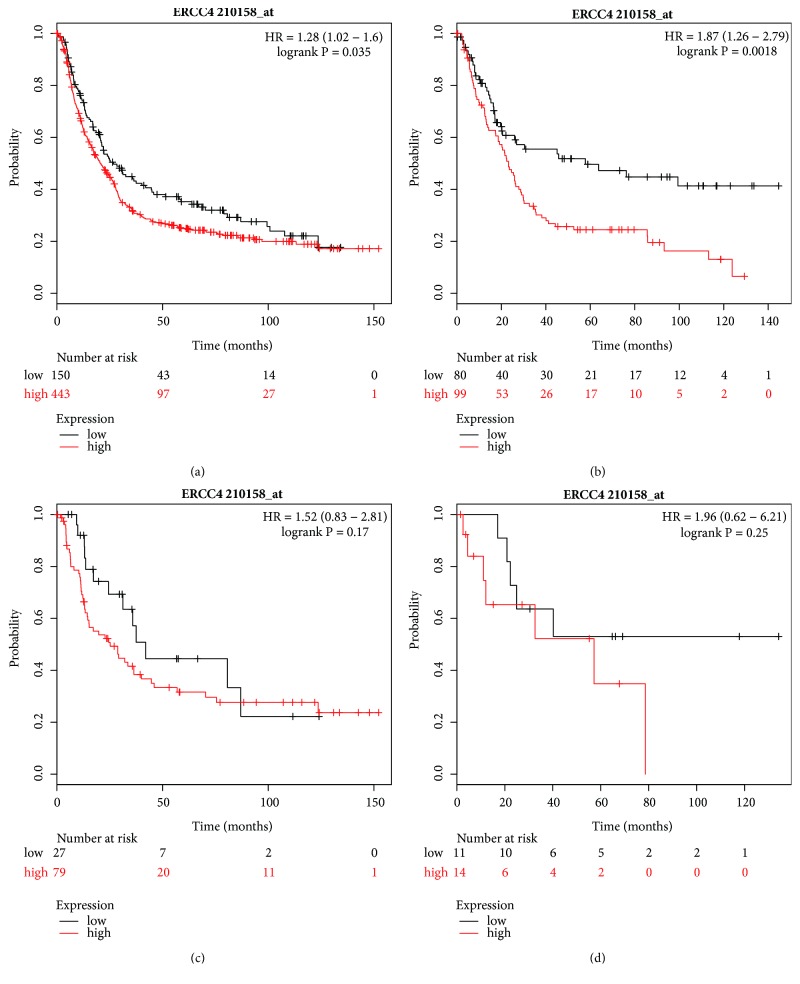
Prognostic value of* ERCC4* (210158_at) expression in the Kaplan–Meier plotter tool. Overall survival curves are plotted for (a) all patients (n=593) and patients with (b) intestinal-type (n=179), (c) diffuse-type (n = 106), and (d) mixed-type (n = 25) gastric cancer.

We then explored the impact of ERCC4 (Affymetrix ID: 210158_at). The high expression of ERCC4 mRNA indicated a worse OS for all GC patients (HR=1.28, 95% CI=1.02–1.6, P=0.035, Figure 7(a)) and for those with intestinal-type GC (HR=1.87, 95% CI=1.26–2.79, P=0.0018, Figure 7(b)), but there was no association between OS for patients with diffuse and mixed-type GC (Figures 7(c) and 7(d)).

**Figure 8:**
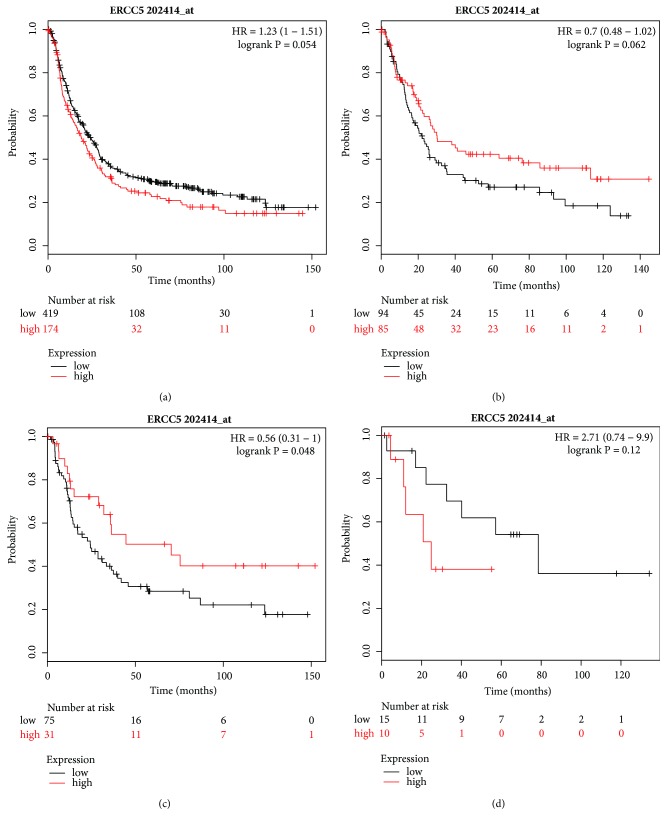
Prognostic value of* ERCC5* (202414_at) expression in the Kaplan–Meier plotter tool. Overall survival curves are plotted for (a) all patients (n=593) and patients with (b) intestinal-type (n=179), (c) diffuse-type patients (n = 106), and (d) mixed-type (n = 25) gastric cancer.

The Affymetrix ID of ERCC5 is 202414_at (Figures 8(a)–8(d)). A high expression of ERCC5 mRNA revealed a favorable OS for patients with diffuse-type GC (HR=0.56, 95% CI=0.31–1, P=0.048, Figure 8(c)), but ERCC5 had no impact on OS and the Lauren classification of GC patients (Figures 8(a), 8(b), and 8(d)).

**Figure 9:**
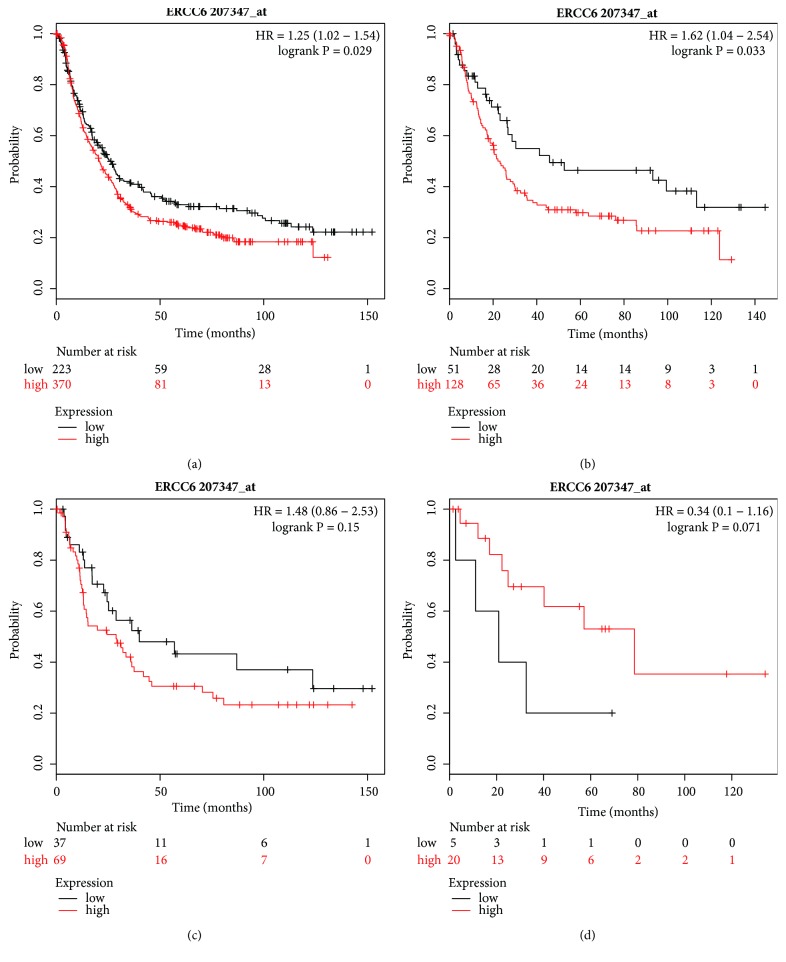
Prognostic value of* ERCC6* (207347_at) expression in the Kaplan–Meier plotter tool. Overall survival curves are plotted for (a) all patients (n=593) and patients with (b) intestinal-type (n=179), (c) diffuse-type (n = 106), and (d) mixed-type (n = 25) gastric cancer.

We next evaluated the diagnostic role of ERCC6 mRNA expression (Affymetrix ID: 207347_at); high ERCC6 mRNA expression was associated with worse OS for all GC patients (HR=1.25, 95% CI=1.02–1.54, P=0.029, Figure 9(a)) and for those with intestinal-type GC (HR=1.62, 95% CI=1.04–2.54, P=0.033, Figure 9(b)), but there was no significant correlation with better or worse OS for patients with diffuse and mixed-type GC (Figures 9(c) and 9(d)).

**Figure 10:**
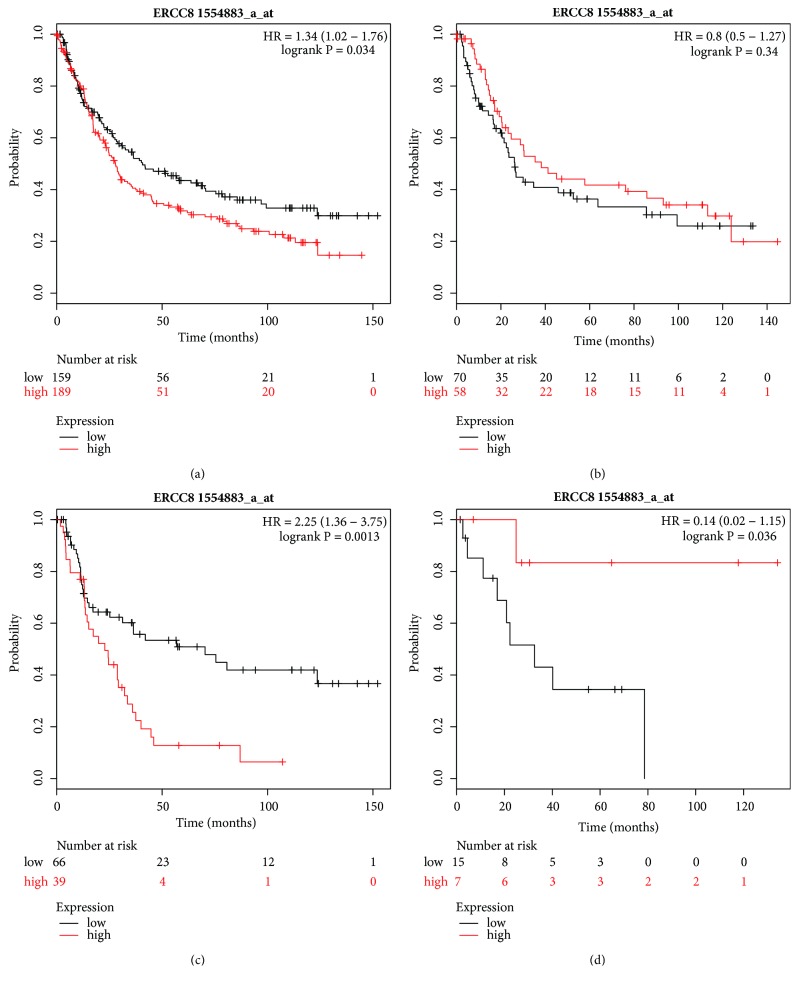
Prognostic value of* ERCC8* (1554883_a_at) expression in the Kaplan–Meier plotter tool. Overall survival curves are plotted for (a) all patients (n=248) and patients with (b) intestinal-type (n=128), (c) diffuse-type (n = 105), and (d) mixed-type patients (n = 22) gastric cancer.

Finally, the Affymetrix ID of ERCC8 is 1554883_at (Figures 10(a)–10(d)). The high expression of ERCC8 mRNA indicated a worse OS for all GC patients (HR=1.34, 95% CI=1.02–1.76, P=0.034, Figure 10(a)) and for those with diffuse-type GC (HR=2.25, 95% CI=1.36–3.75, P=0.0013, Figure 10(c)), but there was no significant correlation with better or worse OS for the patients with intestinal and mixed-type GC (Figures 10(b) and 10(d)).

In addition, the prognostic value of ERCC genes was analyzed further with other clinicopathological characteristics; we stratified analysis of their association with clinical stage (Table 1), differentiation (Table 2), treatment (Table 3), and HER2 status (Table 4).

As presented in Table 1, high ERCC1 and ERCC8 mRNA expression were connected with a better prognosis for patients with Stage 1 GC; ERCC5 and patients with Stage II showed a similar outcome. Conversely, the high expression of ERCC1 mRNA indicated a detrimental prognosis for patients with Stage IV GC; similar outcomes were also observed between ERCC2 and Stage I/IV, ERCC4 and Stage I/II, and ERCC6 and Stage I/II; however, there was no influence on OS for the expression of ERCC3 by clinical stage.

As presented in Table 2, high expression of ERCC3 mRNA was associated with a worse OS for GC patients with well-differentiated, as well as high expression of ERCC4 mRNA effect on moderately differentiated patient and high expression of ERCC6 mRNA effect on poorly/moderately/well-differentiated patient. For the expression of ERCC1 or ERCC2 or ERCC5 or ERCC8, there was no effect on OS in patient with different differentiation.

As shown in Table 3, a high expression of ERCC1 mRNA had a favorable impact on OS for GC patients who received 5-fluorouracil (5-FU)-based adjuvant treatment. On the contrary, the high expression of ERCC3 mRNA was associated with shorter OS for GC patients who received 5-FU-based adjuvant treatment. The high expression of ERCC4 mRNA was associated with shorter OS for GC patients who underwent surgery only, whereas a high expression of ERCC6 mRNA was associated with poor OS for GC patients treated with 5-FU-based adjuvant and surgery. However, the expression of ERCC2, ERCC5, or ERCC8 had no influence on the OS of GC patients in relation to different treatment.

Table 4 shows that the high expression of ERCC1 and ERCC2 mRNA signifies a better OS for HER2-positive GC patients, but high expression of ERCC3 mRNA was associated with poor OS for HER2-positive GC patients; similar results were also observed with ERCC4, ERCC5, and ERCC8.

## 4. Discussion

The caretaker or stability genes, including NER, mismatch repair (MMR), double-strand break repair (DSBR), and base-excision repair (BER) genes, play a vital role in maintaining genomic integrity and stability during normal DNA replication [16]. A wide class of helix-distorting lesions or interstrand adducts induced by exogenous and/or endogenous sources is removed by the NER pathway with the capacity for extreme versatility; otherwise, it would interfere with base pairing and impede normal replication and transcription [17]. ERCC1, ERCC4, and ERCC5 are involved in the repair of interstrand crosslinks and in recombinational DNA repair and DNA damage incision. The ERCC1 and ERCC4 gene products function as partners and encode proteins that interact to produce a nuclease known as the ERCC1-XPF complex [18–21]. ERCC2 and ERCC3 have ATP-dependent DNA helicase activity and function in class II transcription [22, 23]. ERCC6 and ERCC8 are essential for transcription-coupled NER [24, 25]. The outcome of GO and KEGG analysis in the present study indicated that ERCC genes are involved in transcription elongation/initiation from RNA polymerase I/II promoter, termination of RNA polymerase I transcription, NER, DNA duplex unwinding, interstrand cross-link repair, and DNA repair. As shown above, the functions of the ERCC genes are involved in transcription, NER, and DNA duplex unwinding and repair.

Gene and protein interaction networks revealed that the ERCC genes and other relevant genes construct a complex network to interact with each other. Gene–gene interaction networks showed the ERCC gene family are coexpressed with each other, and the PPI network data indicated that ERCC interacted with each other. Coexpression analysis showed that the ERCC gene family indicated a positive correlation between each other. These results suggest that the ERCC gene family could construct a complex cofunction and communicate with each other.

To date, ERCC1 was the most studied of the ERCC gene family in different cancers including GC, colorectal cancer, lung cancer, and ovarian carcinoma. Chang et al. suppressed ERCC1 expression by siRNA-mediated silencing and found that the repair activity of cisplatin-induced DNA damage and cell viability against platinum-based drugs decreased in HeLa S3, MCF-7, and HCT116 cells [26]. Li et al. then found that inhibition of ERCC1 by siRNA made GC cell lines significantly more sensitive to cisplatin (381%) compared with mock controls [27]. However, studies in the relationship between ERCC1 and prognostic value of GC yielded conflicting results. Kim et al. examined samples from 149 patients with advanced GC by immunohistochemistry. They found that patients expressing ERCC1 had a significantly more favorable 5-year OS, and similar results were reported by Bamias et al. [5, 6]. Other studies have observed the opposite results; decreased ERCC1 mRNA levels were associated with a favorable response to 5-FU/cisplatin in patients with primary GC [28]. Yamada et al. analyzed 325 Japanese patients with advanced GC by using real-time reverse transcription polymerase chain reaction (RT-PCR) and found that the response rate to 5-FU for high and low mRNA expression of ERCC1 was 2.7% and 17.5%, respectively (P=0.058). ERCC1 mRNA high expression was correlated with an adverse prognosis [HR 1.37 (1.08–1.75), P = 0.010], but not for therapeutic regimens with cisplatin or 5-FU [29]. In a study by Kwon et al., patients who were examined by immunohistochemistry without ERCC1 expression were more sensitive to 5-FU/oxaliplatin chemotherapy (P = 0.045) and had a longer median OS (P = 0.0396) [30]. Similar results were observed in two meta-analyses [4, 31]. High/positive ERCC1 expression was correlated with worse survival time in head and neck carcinomas, lung cancer, urothelial cancer, and colorectal cancer [32–35]. In our study, high ERCC1 mRNA expression was associated with favorable OS for all GC patients, and especially for patients with Stage 1 GC, 5-FU-based adjuvant treatment, and HER2-positive patients. This was not consistent with most of the research results and may be affected by ERCC4 because of the positive correlation between it and the ERCC1-XPF complex as shown in Figure 3. Another reason could be the limited number of samples. A further prospective study will be needed to clarify this.

Some studies have reported the association between ERCC2 mRNA expression and prognosis in patients with malignant tumors. Zafeer et al. reviewed 132 head and neck cancer patients and assessed tissue samples from 80 patients by PCR. They found that the expression of ERCC2 was associated with the expression of Ki-67 and an aggressive cancer phenotype and concluded that ERCC2 might be used as a biomarker for improved diagnostic and prognostic value in head and neck cancer. [36]. Huang et al. analyzed ERCC2 expression by immunohistochemistry in 180 colorectal cancer patients with adjuvant chemotherapy and showed that ERCC2 was not related to survival time; moreover, similar results were observed in a study by Kassem et al. [8, 37]. As far as GC is concerned, the prognosis associated with ERCC2 mRNA expression has not been reported. In our present study, a high expression of ERCC2 mRNA was markedly associated with a favorable OS in general and in particular for HER2 positive GC patients but was correlated with poor OS for patients with intestinal-type and Stage I/IV GC. Owing to this paradoxical result, we cannot conclude that ERCC2 can be used for prognosis; the inconsistent results need to be resolved in further research.

ERCC3 is needed for transcription and DNA repair. In a situation where DNA is damaged, XPB advances NER by unwinding the dsDNA surrounding a DNA lesion [38]. The clinical function of ERCC3 is clearly known to be associated with inherited disease including UV-hypersensitive NER syndromes* xeroderma pigmentosum *(XP), Cockayne Syndrome (CS), combined XP and CS (XP/CS), and trichothiodystrophy (TTD) [39–42]. At present, few studies have reported the prognosis capability of ERCC3 mRNA expression in malignant tumors, and there are no reports in GC. Terashita et al. analyzed 43 samples from patients with primary esophageal squamous cell carcinomas by using RT-PCR and found that low ERCC3 mRNA expression indicated tumor progression and a shorter postoperative survival time [43]. Apart from a high expression of ERCC3 mRNA indicating a worse OS for patients with well-differentiated GC, our present study revealed no association between ERCC3 and OS.

Napieralski et al. analyzed 61 neoadjuvant treated GC patients and found that the high expression of ERCC4 mRNA showed a trend for a correlation with shortened survival (P=0.10) [44]. However, the association of ERCC4 with the prognosis of other cancers was controversial. Alexander et al. reported that ERCC4 expression was not associated with OS in esophageal cancer [45]. Liu et al. found that ERCC4 high expression was associated with a longer OS in male patients with colon cancer (HR=0.54, 95% CI=0.30–0.96), but not in all patients and rectal cancer patients [46]. Conversely, Vaezi et al. analyzed samples from 80 patients with head and neck squamous cell carcinoma by immunohistochemistry and revealed that high ERCC4 expression was correlated with earlier time to progression; one-year progression-free survival for high expression was 47% compared to 72% for low expression [47]. In our present study, high ERCC4 mRNA expression was associated with worse OS for all GC patients, in particular for intestinal-type and Stage I/II GC, moderately differentiation, patients with surgery only treatment, and HER2 positive GC patients. Except for diffuse- and mixed-type patients, the results were consistent with a study by Napieralski et al., suggesting that ERCC4 may be a negative prognostic biomarker.

There were some reports on the prognosis of ERCC5 mRNA expression in malignant tumors, but none in GC. Walsh et al. reported a longer progression-free survival in ovarian cancer patients with a downregulation of ERCC5 gene expression [48]. Liu et al. analyzed colorectal cancer by ONCOMINE and found that ERCC5 expression was associated with deeper T stage and distant metastasis but was not associated with OS. Italiano et al. reported that high ERCC5 mRNA expression has a significantly longer median progression-free survival (13.7 months vs 1.7 months) in breast cancer [49]. In our present study, high expression of ERCC5 mRNA was only associated with longer OS for diffuse-type GC patients, but there was no impact on OS for other patients and other Lauren classifications. Therefore, ERCC5 cannot predict the prognosis of GC.

The study of the prognosis of ERCC6 mRNA expression has been limited to the malignancy of tumors. Zhao et al. downregulated ERCC6 expression by using short hairpin RNA to enhance the sensitivity of HCT116 and DLD1 cells to 5-FU and found that ERCC6 mRNA high expression revealed a worse OS in 38 pairs of colorectal cancers with or without 5-FU treatment [50]. In our present study, ERCC6 mRNA high expression was associated with shorter OS for all GC patients, especially for those with intestinal-type GC, patients with Stage I/II, poorly/moderately/well patients, and patients receiving 5-FU-based adjuvant/surgery only treatment. This was in accordance with a study by Zhao et al., although not in relation to GC. A possible explanation for these results was that ERCC6 increased the chemoresistance of GC.

Several studies have reported a relationship between ERCC8/ERCC6 and the pathogenesis of CS; however, the prognosis ability or mRNA expression of ERCC8 in cancer is seldom reported. Zhao et al. analyzed the expression of ERCC8 mRNA using the KM plotter database and found that ERCC8 high expression was connected with poorer OS in all ovarian cancer patients, and also in patients with clinical stages III and IV [51]. In our present study, the high expression of ERCC8 mRNA indicated a worse OS for all GC patients, especially for those with diffuse-type GC and for HER2-positive patients, suggesting that ERCC8 may be a passive prognostic biomarker. Inconsistently, high expression of ERCC8 was correlated with favorable prognosis for patients with Stage 1; a possible reason for this was the shortage of samples.

We need to recognize that our present study has the following limitations. First, the clinical parameter which was extracted from the public dataset was incomprehensive; therefore, the relationship between expression of ERCC genes and OS in GC patients was analyzed by univariate statistics in our study. Second, the joint effect of ERCC gene expression in GC prognosis cannot be analyzed by the KM plotter. Third, the KM plotter data was from multiple databases and the samples were probably collected at different places using different protocols. Therefore, despite scientific and reliable methods for data merger, batch effects between these raw data still may have some impact on the results. Fourth, because the KM plotter data was from the GEO database, and all the data were generated from the genome-wide expression profiling chip, the present study cannot investigate the relationship between ERCC protein level and GC prognosis.

Even with these limitations, our data is the first to investigate the relationship between expression of ERCC genes and OS in GC patients, as well as the prognostic roles in different strata of GC. Therefore, these findings reveal the perceptiveness of ERCC genes in the clinical outcome of GC and may provide an application for clinical decisions in GC.

## 5. Conclusion

In summary, our findings have demonstrated that a high expression of ERCC4 and ERCC6 mRNA was significantly associated with worse OS for all patients with GC and in particular those with intestinal-type GC. In addition, ERCC8 mRNA high expression was significantly associated with worse OS for all patients with GC and in particular for those with diffuse-type GC. ERCC4, ERCC6, and ERCC8 could be potential biomarkers for the prognosis of GC patients; moreover, they also could serve as potential therapeutic targets. However, our results need further investigation.

## Figures and Tables

**Table 1:** The prognostic value of the mRNA expression of ERCC in different clinical stage GC patients.

Genes	Stage	cases	HR (95% CI)	P-value
ERCC1	I	39	0.31 (0.1–0.94)	0.029
	II	49	1.54 (0.62–3.78)	0.35
	III	217	0.83 (0.6–1.15)	0.26
	IV	77	2.64 (1.48–4.71)	0.00068
ERCC2	I	39	3.17 (0.96–10.44)	0.046
	II	49	0.57 (0.21–1.55)	0.26
	III	217	1.35 (0.95–1.91)	0.096
	IV	74	1.89 (1.06–3.36)	0.028
ERCC3	I	39	2.68 (0.73–9.87)	0.12
	II	49	0.46 (0.19–1.07)	0.065
	III	217	1.25 (0.9–1.75)	0.19
	IV	74	0.75 (0.43–1.32)	0.32
ERCC4	I	39	5.03 (1.11–22.74)	0.02
	II	49	3.29 (139.–7.77)	0.0043
	III	217	1.45 (0.98–2.13)	0.063
	IV	74	0.72 (0.41–1.27)	0.25
ERCC5	I	39	0.29 (0.06–1.32)	0.088
	II	49	0.36 (0.13–0.99)	0.038
	III	217	1.27 (0.87–1.85)	0.22
	IV	74	0.57 (0.31–1.04)	0.065
ERCC6	I	39	7.09 (0.91–55.24)	0.03
	II	49	2.84 (1.22–6.62)	0.012
	III	217	1.23 (0.86–1.76)	0.26
	IV	74	0.62 (0.35–1.09)	0.095
ERCC8	I	34	0.23 (0.06–0.83)	0.015
	II	44	1.68 (0.65–4.34)	0.28
	III	109	1.46 (0.91–2.36)	0.12
	IV	66	0.66 (0.37–1.2)	0.17

*Notes*. ERCC, excision repair cross-complementing; GC, gastric cancer; HR, hazard ratio; CI, confidence interval.

**Table 2:** The prognostic value of the mRNA expression of ERCC in different differentiation GC patients.

Genes	Differentiation	cases	HR (95% CI)	P-value
ERCC1	Poorly	165	0.7 (0.45–1.07)	0.099
	Moderately	67	1.75 (0.91–3.36)	0.09
	Well	32	1.87 (0.55–6.38)	0.31
ERCC2	Poorly	165	1.49 (0.91–2.43)	0.11
	Moderately	67	1.67 (0.85–3.3)	0.13
	Well	32	1.7 (0.68–4.23)	0.25
ERCC3	Poorly	165	1.37 (0.92–2.03)	0.12
	Moderately	67	1.39 (0.67–2.9)	0.38
	Well	32	4.15 (1.47–11.69)	0.004
ERCC4	Poorly	165	1.48 (0.94–2.33)	0.089
	Moderately	67	2.21 (1.12–4.34)	0.019
	Well	32	2.2 (0.65–7.49)	0.2
ERCC5	Poorly	165	1.26 (0.85–1.88)	0.25
	Moderately	67	0.6 (0.29–1.21)	0.15
	Well	32	1.37 (0.58–3.25)	0.47
ERCC6	Poorly	165	1.61 (1.01–2.55)	0.042
	Moderately	67	2.02 (1.05–3.88)	0.032
	Well	32	4.09 (0.95–17.66)	0.041
ERCC8	Poorly	121	1.5 (0.93–2.44)	0.097
	Moderately	67	0.53 (0.28–1.03)	0.057
	well	5		

*Notes*. ERCC, excision repair cross-complementing; GC, gastric cancer; HR, hazard ratio; CI, confidence interval.

**Table 3:** The prognostic value of the mRNA expression of ERCC in different treatment GC patients.

Genes	Treatment	cases	HR (95% CI)	P-value
ERCC1	surgery	174	1.29 (0.85–1.96)	0.22
	5-Fu	153	0.49 (0.33–0.72)	0.00023
ERCC2	surgery	174	0.84 (0.55–1.27)	0.4
	5-Fu	153	1.37 (0.95–1.99)	0.092
ERCC3	surgery	174	1.26 (0.82–1.92)	0.29
	5-Fu	153	2.2 (1.5–3.22)	0.00035
ERCC4	surgery	174	1.8 (1.19–2.74)	0.0049
	5-Fu	153	1.41 (0.99–1.99)	0.053
ERCC5	surgery	174	0.78 (0.5–1.21)	0.26
	5-Fu	153	1.28 (0.9–1.81)	0.16
ERCC6	surgery	174	1.7 (1.03–2.79)	0.035
	5-Fu	153	1.49 (1.04–2.12)	0.028
ERCC8	surgery	174	1.41 (0.92–2.14)	0.11
	5-Fu	34	2.25 (0.79–6.4)	0.12

*Notes*. ERCC, excision repair cross-complementing; GC, gastric cancer; HR, hazard ratio; CI, confidence interval.

**Table 4:** The prognostic value of the mRNA expression of ERCC in HER2 status GC patients.

Genes	*HER2 status*	cases	HR (95% CI)	P-value
ERCC1	negative	298	0.75 (0.56–1.01)	0.054
	positive	295	0.71 (0.51–0.98)	0.039
ERCC2	negative	298	0.76 (0.56–1.03)	0.073
	positive	295	0.72 (0.54–0.95)	0.022
ERCC3	negative	298	0.8 (0.59–1.09)	0.15
	positive	295	1.45 (1.1–1.92)	0.0075
ERCC4	negative	298	1.27 (0.94–1.72)	0.11
	positive	295	1.52 (1.13–2.05)	0.0059
ERCC5	negative	298	0.86 (0.64–1.14)	0.29
	positive	295	1.45 (1.1–1.9)	0.0082
ERCC6	negative	298	1.22 (0.92–1.62)	0.18
	positive	295	1.27 (0.94–1.71)	0.11
ERCC8	negative	195	0.79 (0.54–1.15)	0.21
	positive	153	1.8 (1.19–2.74)	0.0052

*Notes*. ERCC, excision repair cross-complementing; GC, gastric cancer; HR, hazard ratio; CI, confidence interval.

## Data Availability

The data used to support the findings of this study are included within the article.
